# Cooking fuel choices and garbage burning practices as determinants of birth weight: a cross-sectional study in Accra, Ghana

**DOI:** 10.1186/1476-069X-11-78

**Published:** 2012-10-17

**Authors:** Adeladza K Amegah, Jouni JK Jaakkola, Reginald Quansah, Gameli K Norgbe, Mawuli Dzodzomenyo

**Affiliations:** 1 Department of Human Biology, School of Biological Sciences, University of Cape Coast, Cape Coast, Ghana; 2 Center for Environmental and Respiratory Health Research, University of Oulu, Oulu, Finland; 3 Department of Population, Family and Reproductive Health, School of Public Health, University of Ghana, Legon, Accra, Ghana; 4 Department of Biological, Occupational and Environmental Health, School of Public Health, University of Ghana, Legon, Accra, Ghana

**Keywords:** Indoor air pollution, Birth weight, Cooking fuel, Garbage burning

## Abstract

**Background:**

Effect of indoor air pollution (IAP) on birth weight remains largely unexplored but yet purported as the most important environmental exposure for pregnant women in developing countries due to the effects of second-hand smoke. We investigated the associations between the determinants of indoor air quality in households and birth weight.

**Methods:**

A cross-sectional study of 592 mothers and their newborns using postnatal services at the Korle Bu Teaching Hospital located in Accra, Ghana was conducted in 2010 to collect information on characteristics of indoor environment and other potential determinants of fetal growth. Birth weight was recorded from hospital records.

**Results:**

Household cooking fuel choices and garbage burning practices were determinants of birth weight. Multivariate linear regression analysis adjusting for age, social class, marital status and gravidity of mothers, and sex of neonate resulted in a 243g (95% CI: 496, 11) and 178g (95% CI: 421, 65) reduction in birth weight for use of charcoal, and garbage burning respectively compared with use of LPG only. The estimated reductions in birth weight was not statistically significant. Applying the ordinal scale exposure parameter nonetheless revealed a significant exposure-response relationship between maternal exposures from charcoal use and garbage burning, and birth weight. Generalized linear models adjusting for confounders resulted in a 41% (risk ratio [RR] = 1.41; 95% CI: 0.62, 3.23) and 195% (RR=2.95; 95% CI: 1.10, 7.92) increase in the risk of low birth weight (LBW) for use of charcoal, and garbage burning respectively compared with use of LPG only. A combination of charcoal use and household garbage burning during pregnancy on fetal growth resulted in a 429g (95% CI: 259, 599) reduction in birth weight and 316% (RR=4.16; 95% CI: 2.02, 8.59) excess risk of LBW. Sensitivity analysis performed by restricting the analysis to term births produced similar results.

**Conclusions:**

Maternal use of charcoal as a cooking fuel during pregnancy and burning of garbage at home are strong determinants of average fetal growth and risk of LBW. Efforts to reduce maternal exposures to IAP are thus important to improve birth outcomes.

## Background

Birth weight is an important determinant and predictor of neonatal and infantile growth and survival, as well as health in childhood and later life. Low birth weight (LBW), defined by the World Health Organization (WHO) as birth weight less than 2500 grams [1] is closely associated with neonatal and infant mortality and morbidity, reduced growth, impaired immune function and poor cognitive development [2]. LBW has also been associated with childhood and adult disorders such as asthma [3], type 2 diabetes, hypertension, and coronary heart disease in many studies [4,5].

Birth weight is determined by multiple factors with Sohl and Moore [6] estimating heredity and environmental factors to account for 40% and 60% of birth weight respectively. Stephenson and Symonds [7] have also suggested that about 60% of the variation in birth weight can be explained by environmental factors. Environmental factors are therefore clearly important determinants of birth weight. Spencer and Logan [8] note that secular changes in birth weight, birth weight variations within genetically similar populations, and birth weight depiction of a reverse social gradient such that increasing disadvantage is associated with decreasing birth weight all suggest an environmental influence.

The maternal household environment has been proposed to have a strong influence on birth weight. Stephenson and Symonds [7] identified the general and immediate maternal environment to account for almost 40% of the variation in birth weight attributable to environmental factors. Cooking fuels such as charcoal and wood, and garbage burning are important sources of indoor air pollution in maternal households with poor ventilation of homes often worsening indoor air pollution. The relationship between indoor air pollution (IAP) and birth weight remains largely unexplored with studies in Guatemala [9,10], Zimbabwe [11], Pakistan [12] and India [13,14] that reported association of biomass fuel use with reduced and low birth weight identified to be the only studies to have examined this relationship.

The apparent lack of research on the link between indoor air pollution and birth weight is totally at variance with the widespread projection of indoor air pollution as the most important environmental exposure for pregnant women especially in developing countries due to the effects of second-hand smoke. It is against this background that our objective was to study the associations between the determinants of indoor air quality such as cooking fuel choices, cooking sequence and patterns, and household garbage burning practices, and birth weight in Accra households.

## Methods

### Study design and site

A cross-sectional study was conducted among mothers and newborns of the Maternity Department of the Korle Bu Teaching Hospital (KBTH). KBTH is located in the south-western part of Accra and serves as the national referral centre for southern Ghana. The catchment area of KBTH's Maternity Department are communities in the south-western corridor of Accra. The comprehensive and specialist services on offer at KBTH as a whole sees majority of mothers residing in the south-western part of Accra preferring this facility to others in the area that equally provides Reproductive and Child Health (RCH) services. Mothers from other parts of Accra and surrounding areas also access RCH services at KBTH for the very same reason or because they have been referred for an underlying health risk.

### Study population and sampling procedure

The source population comprised all nursing mothers residing in Accra. Six hundred and forty seven mothers that had singleton deliveries with no gross anatomical deformities at KBTH Maternity Department and accessing postnatal services at the same facility were randomly sampled from a shortlist provided by the department's Biostatistics Unit. Selected mothers who visited the postnatal clinic were interviewed after verification that they were non- referral patients, and resided in Accra and their respective neighbourhood throughout the duration of the pregnancy. The study population included 592 newborns (response rate 91.5%).

### Exposure assessment

We studied the independent and joint effects of charcoal usage and garbage burning on fetal growth. Exposure information was collected by using a structured questionnaire to obtain information on the type of cooking fuel used, type and ventilation rating of cooking enclosure, frequency and duration of cooking activities, amount of time spent in cooking area during cooking sessions, practice of garbage burning in household, frequency of garbage burning, and presence of mother during garbage burning sessions.

Households using liquefied petroleum gas (LPG) only without charcoal or garbage burning constituted the reference category. The two main household fuel types were used alone and in combination. To study the independent and joint effects of these together with garbage burning we used the following exposure categories: (1) charcoal use only, (2) charcoal and LPG use, (3) garbage burning only, (4) both LPG use and garbage burning, and (5) both charcoal use and garbage burning. For charcoal and garbage burning we also used ordinal scale exposure parameter: low, moderate and high.

The level of exposure to charcoal and garbage burning was defined as follows. For use of charcoal only, step one involved (a) classifying the following as high exposure practices: cooking up to the seventh or ninth month of pregnancy, cooking frequency of four or more times per week, staying in cooking area throughout the whole duration of each cooking session, and cooking area ventilation ratings of poor or satisfactory, and (b) classifying the following as low exposure practices: cooking up to the sixth month of pregnancy, cooking frequency of less than four times per week, staying in cooking area for up to about half the duration of each cooking session, and cooking area ventilation ratings of good, very good or excellent. Step two involved (a) classifying maternal report of all four high exposure practices or a combination of any three high exposure practices and any one low exposure practice as high exposure; (b) classifying maternal report of a combination of any two high exposure practices and any two low exposure practices as moderate exposure, and (c) classifying maternal report of all four low exposure practices or a combination of any three low exposure practices and any one high exposure practices as low exposure.

With regards to garbage burning only, step one involved (a) classifying the following as high exposure practices: garbage burning frequency of four or more times per week, and regular presence in household during combustion, and (b) classifying the following as low exposure practices: garbage burning frequency of less than four times per week, and occasional presence in household during combustion. Step two involved (a) classifying maternal report of the two high exposure practices as high exposure, (b) classifying maternal report of a combination of any one high exposure practice and any one low exposure practice as moderate exposure, and (c) classifying maternal report of the two low exposure practices as low exposure.

### Outcomes

The main outcome was fetal growth which was measured both as birth weight in grams and low birth weight (birth weight below 2500 grams). Birth weight of the newborns was obtained from hospital records. An RGZ-20 baby scale which measures birth weight in kilograms up to two decimal places was used at the facility to weigh newborns immediately after birth. The scale was regularly calibrated by the health staff.

### Ethical considerations

Ethical approval was sought for the study from Ghana Health Service Ethical Review Committee. Informed consent form was used to seek the consent of all participants before inclusion in the study.

### Statistical analysis

We compared the average birth weight and the risk of low birth weight according to categories of charcoal and garbage burning-related exposure using *t*-test and Chi-square test to assess the role of chance. Chi-square test was also used to investigate the differences in cooking fuel choices of mothers according to their socioeconomic characteristics. We applied multivariate methods for assessing the exposure-outcome relations. First, we applied multiple linear regression to estimate the independent and joint effects of the two exposures on average birth weight. Second, we applied generalized linear models (PROC GENMOD) with Poisson distribution and log link to estimate the independent and joint effects of indoor air pollution on the risk of LBW. All models were adjusted for age, social class, marital status and gravidity of mothers; and sex of neonate. Pearson's correlation and Mantel-Haenszel linear by linear Chi-square test was used for trend analysis of the exposure-response relationships. We performed sensitivity analysis by restricting the analysis to term births (≥37 weeks of gestation). SPSS version 16.0 was used to perform all the analysis with the exception of the PROC GENMOD analysis which was done with SAS version 9.3.

## Results

The characteristics of the mothers and their neonates are presented in Table 1 and Table 2. More than half (58.8%) of the respondents were classified as low social class with about 5% of the respondents identified as high social class. A quarter (25.3%) of the respondents were young mothers (<24 years) with older mothers (>35 years) making up about 15% of the respondents studied. Majority of the respondents (77.2%) were married. A high proportion of the respondents (45.1%) were educated up to junior high level. About 7% of the respondents had no formal education. Majority of the respondents (43.6%) were traders and street vendors. Hairdressers and seamstresses made up about 30% of the respondents studied. The proportion of office workers was 6%. About 39% of the respondents were primigravida. Of the mothers studied, 94.3% were cooking during pregnancy. Half of the mothers who cooked (50.5%) used charcoal only with about 29% of them using LPG only. About 19% of the mothers used a combination of both fuels. Cooking fuel choices of mothers was dependent on their social class, educational level and occupation. Of the proportion of charcoal users, and charcoal & LPG users, more than half respectively were identified as low social class. Of the proportion of mothers identified as high social class, majority used LPG for cooking. Also, of the proportion of mothers with no formal education and those educated up to junior high level, over half of them used charcoal for cooking. Of the tertiary educated mothers, over half of them used LPG for cooking. Charcoal users were mostly traders, street vendors, hairdressers and seamstresses. Office workers mostly used LPG for cooking. About 27% of the mothers studied reported garbage burning in their homes during pregnancy. The proportion of mothers using charcoal only for cooking and burning garbage at home simultaneously was 15%.

**Table 1:** Characteristics of the Study Population

	**Total N=592**	**Reference**	**Exposure categories**				
		LPG use^*^N=161	Charcoal use only^*^N=282	Charcoal use and LPGuse^*^N=104	**Garbage burning only N=160**	**LPG use and garbage burning N=30**	**Charcoal use and garbage burning N=90**
**Age Group (years)**							
< 20	5.7	2.5	6.4	7.7	3.8	0.0	4.4
20-29	50.0	46.0	53.5	48.1	47.5	40.0	47.8
30-39	42.3	50.9	37.6	42.3	46.2	56.7	45.6
> 39	2.0	0.6	2.5	1.9	2.5	3.3	2.2
**Social Class**							
Low	58.8	43.5	66.0	63.5	56.2	43.3	57.8
Middle	36.1	46.6	31.6	33.7	39.4	40.0	40.0
High	5.1	9.9	2.5	2.9	4.4	16.7	2.2
**Marital Status**							
Married	77.2	90.1	74.1	72.1	76.9	90.0	77.8
Unmarried	22.8	10.0	25.9	27.9	23.1	10.0	22.2
**Educational Level**							
Primary	15.2	8.7	18.8	17.3	16.9	13.3	14.4
Junior High	45.1	39.8	51.4	37.5	45.6	36.7	51.1
Senior High	25.3	34.8	16.0	36.5	28.1	40.0	23.3
Tertiary	7.1	15.5	3.5	3.8	5.6	10.0	5.6
None	7.3	1.2	10.3	4.8	3.8	0.0	5.6
**Occupation**							
Trader/Street vendor	43.6	33.5	50.7	41.3	47.5	36.7	51.1
Fish monger/Caterer	5.9	7.5	4.6	7.7	3.8	6.7	2.2
Hairdresser/Seamstress	29.7	29.8	27.0	33.7	31.9	36.7	30.0
Office worker	5.9	13.0	2.8	5.8	5.6	16.7	3.3
Housewife/Unemployed	9.8	6.8	12.1	4.8	6.9	0	10.0
Other	5.1	9.3	2.8	6.7	4.4	3.3	3.3
**Gravidity**							
Primigravida	39.2	41.0	36.9	36.5	33.1	23.3	36.7
Multigravida	60.8	59.0	63.1	63.5	66.9	76.7	63.3

Values reported in table are percentages.

^*^X^2^ Test for differences in cooking fuel choices: social class, p<0.0001; educational level, p<0.0001, occupation, p<0.0001.

**Table 2:** Characteristics of Neonates, and LBW Cases and Mean Birth Weight (grams) by Cooking Fuel Use and Garbage Burning at Home

	**Total N=592**	**Reference**	**Exposure categories**				
		**LPG use N=161**	**Charcoal use only N=282**	**Charcoal use and LPG use N=104**	**Garbage burning only N=160**	**LPG use and garbage burning N=30**	**Charcoal use and garbage burning N=90**
**Sex**							
Male	53.2						
Female	46.8						
**Birth Order**							
1	38.3						
2-3	45.9						
4-5	13.5						
6+	2.2						
Mean^*^± SD	2949±634	3165±540	2843±650	2911±613	2855±690	3040±703	2773±721
LBW^**^n(%)	109(18.4)	15(9.3)	64(22.7)	20(19.2)	37(23.1)	6(20.0)	23(25.6)

Note. *SD* Standard Deviation. Values reported for sex and birth order of neonates are percentages.

^*^t-Test for differences in mean birth weight: LPG use vs. Charcoal use, p<0.0001; LPG use vs. Charcoal use & LPG use, p<0.0001; LPG use vs. Garbage burning, p<0.0001; LPG use vs. LPG use & garbage burning, p=0.162; LPG use vs. Charcoal use & garbage burning, p<0.0001.

^**^X^2^ Test for differences in LBW cases: all fuel type categories, p=0.002; LPG use vs. Charcoal use, p<0.0001; LPG use vs. Charcoal use & LPG use, p=0.020; LPG use vs. Garbage burning, p<0.0001; LPG use vs. LPG use & garbage burning, p=0.026; LPG use vs. Charcoal use & garbage burning, p<0.0001.

More than half of the neonates (53.2%) were males. First order births made up about 38% of the neonates. Neonates delivered to charcoal users, and charcoal & LPG users were respectively 322g and 254g lighter than neonates delivered to LPG users. The differences in means were highly significant. Also, neonates delivered to mothers burning garbage at home were 310g lighter than neonates delivered to LPG users. The mean difference was also significant. Neonates delivered to mothers using charcoal only for cooking as well as burning garbage at home were 392g lighter than neonates delivered to LPG user. The mean difference was statistically significant. About 18% of the neonates were born LBW. The risk of LBW was related to fuel choices and garbage burning practices of mothers, independently and jointly.

The unadjusted and adjusted effect of cooking fuel use and garbage burning at home on birth weight and risk of LBW are presented in Table 3 and Table 4. Charcoal use and garbage burning at home were all determinants of reduced birth weight. The estimated reduction in birth weight was 243g (95% CI: 496, 11) and 178g (95% CI: 421, 65) for use of charcoal, and garbage burning at home. The associations were however not statistically significant. An exposure-response relationship was nonetheless noted with high exposure from charcoal use and garbage burning associated with an increased reduction in birth weight. A linear trend test of the association was statistically significant. The use of charcoal together with garbage burning at home was associated with further reductions in birth weight. The estimated reduction in birth weight was 429g (95% CI: 259, 599).

**Table 3:** Unadjusted and Adjusted Effect of Cooking Fuel Use and Garbage Burning at Home

**Exposure category**	**All births (N=592)**		**Term births (N=442)**	
	**Unadjusted β (95% CI)**	**Adjusted β (95% CI)**	**Unadjusted β (95% CI)**	**Adjusted β (95% CI)**
**Charcoal use only (n=282)**	−267 (−518, -15)	−243 (−496, 11)	−136 (−399, 127)	−108 (−372, 155)
Low (n=40)	−237 (−450, -24)	−262 (−477, -47)	−205 (−426, 15)	−181 (−403, 42)
Moderate (n=106)	−300 (−451, -150)	−289 (−442, -137)	−165 (−323, -7)	−144 (−305, 16)
High (n=136)	−363 (−503, -223)	−381 (−523, -239)	−274 (−419, -130)	−278 (−425, -131)
	*Trend p value = 0.000*		*Trend p value = 0.000*	
**Charcoal use and LPG use**	−146 (−441, 149)	−109 (−406, 188)	−114 (−416, 189)	−82 (−385, 220)
**Garbage burning only (n=160)**	−153 (−395, 88)	−178 (−421, 65)	−114 (−361, 134)	−133 (−382, 116)
Low (n=57)	−144 (−322, 44)	−140 (−331, 50)	−11 (−205, 183)	9 (−188, 205)
Moderate (n=60)	−488 (−673, -304)	−489 (−676, -302)	−389 (−588, -191)	−403 (−602, -204)
High (n=43)	−386 (−594, -178)	−383 (−596, -170)	−247 (−463, -31)	−217 (−438, 3)
	*Trend p value = 0.000*		*Trend p value = 0.001*	
**LPG use and garbage burning**	−153 (−396, 90)	−169 (−415, 77)	−114 (−361, 134)	−126 (−375, 123)
**Charcoal use and garbage burning**	−420 (−584, -255)	−429 (−599, -259)	−249 (−427, -72)	−245 (−428, -62)

Note. *CI* Confidence interval, Effect estimate (β) is in grams.

Trend test is for charcoal use only and garbage burning only exposure categories.

LPG users (n=161) served as reference category for all exposure categories.

Effect estimates adjusted for age, social class, marital status and gravidity of mothers, and sex of neonate.

**Table 4:** Unadjusted and Adjusted Risk of Low Birth Weight attributable to Cooking Fuel Use and Garbage Burning at Home

**Exposure category**	**All births (N=592)**		**Term births (N=442)**	
	**Unadjusted RR (95% CI)**	**Adjusted RR (95% CI)**	**Unadjusted RR (95% CI)**	**Adjusted RR (95% CI)**
**Charcoal use only (n=282)**	1.28 (0.58, 2.84)	1.41 (0.62, 3.23)	1.10 (0.39, 3.12)	1.14 (0.39, 3.35)
Low (n=40)	2.42 (1.14, 5.11)	2.89 (1.34, 6.21)	2.12 (0.78, 5.73)	2.05 (0.76, 5.56)
Moderate (n=106)	2.63 (1.46, 4.73)	2.70 (1.51, 4.84)	1.71 (0.75, 3.91)	1.59 (0.72, 3.53)
High (n=136)	2.29 (1.28, 4.09)	2.41 (1.34, 4.35)	1.88 (0.89, 4.01)	1.79 (0.81, 3.94)
	*Trend p value = 0.003*		*Trend p value = 0.112*	
**Charcoal use and LPG use**	0.97 (0.37, 2.58)	1.09 (0.41, 2.93)	1.07 (0.32, 3.54)	1.05 (0.31, 3.59)
**Garbage burning only (n=160)**	2.91 (1.12, 7.56)	2.95 (1.10, 7.92)	2.75 (0.84, 8.99)	2.77 (0.81, 9.52)
Low (n=57)	2.30 (0.96, 5.49)	2.50 (1.03, 6.04)	1.53 (0.46, 5.17)	1.56 (0.45, 5.42)
Moderate (n=60)	3.88 (1.82, 8.28)	4.32 (2.03, 9.20)	3.71 (1.41, 9.74)	3.95 (1.50, 10.42)
High (n=43)	4.06 (1.84, 8.97)	4.59 (2.01, 10.48)	3.61 (1.31, 9.96)	3.59 (1.20, 10.77)
	*Trend p value = 0.000*		*Trend p value = 0.002*	
**LPG use and garbage burning**	2.91 (1.12, 7.56)	2.80 (1.04, 7.54)	2.75 (0.84, 8.99)	2.60 (0.76, 8.88)
**Charcoal use and garbage burning**	3.72 (1.81, 7.66)	4.16 (2.02, 8.59)	3.03 (1.18, 7.76)	3.06 (1.15, 8.14)

Note. *RR* Risk ratio, *CI* Confidence interval.

Trend analysis is for charcoal use only and garbage use only exposure categories.

LPG users (n=161) served as reference category for all exposure categories.

Risk ratios adjusted for age, social class, marital status and gravidity of mothers, and sex of neonate.

Use of charcoal and garbage burning at home were risk factors for LBW. Garbage burning at home resulted in a 195% increase in the risk of LBW. High exposure from garbage burning was also associated with a 359% increase in the risk of LBW. A linear trend test of the association was statistically significant. Charcoal use generally was associated with a small and statistically insignificant increase in the risk of LBW. Applying the ordinal scale exposure parameter for charcoal however resulted in a statistically significant association, albeit an inverse trend was observed with high exposure associated with decrease risk. Charcoal use together with garbage burning at home was associated with further increase in the risk of LBW. The estimated increase in the risk of LBW for joint charcoal use and garbage burning was 316%.

The sensitivity analysis produced similar results but with slightly lower effect estimates generally.

## Discussion

The results of our study indicate a strong, exposure-related inverse association between maternal use of charcoal as cooking fuel during pregnancy and birth weight of the newborn. The average birth weight of babies born among exposed mother was 243g (95% CI: 496, 11) lower compared with the babies of mothers using LPG as cooking fuel. Garbage burning at home was an important risk factor for LBW. Garbage burning was associated with a 195% (RR=2.95; 95% CI: 1.10, 7.92) increase in the risk of LBW. Joint evaluation of these two exposures resulted in further reductions in birth weight and additional increase in the risk of LBW.

### Validity of results

We selected consecutive mothers giving birth in a teaching hospital and thus the study population represents a defined catchment area. We achieved a high response rate (91.5%) which minimizes selection bias. Mothers giving birth at KBTH as against other facilities in the study area where not a distinct cohort from the source population but do so mainly because of the hospital's proximity to their homes and/or the comprehensive and specialist services on offer. We also carefully excluded mothers referred to the facility for whatever reason from the study. The outcome of interest was measured and recorded independently from the study and represent a well defined and objective variable with a negligible measurement error. Information on exposure and potential confounders was collected retrospectively. Use of cooking fuel choices and garbage burning practices represents quantitatively well-defined entity and it is reasonable to assume that retrospective data collection resulted in relatively reliable data on maternal exposure during pregnancy. The ordinal scale exposure variable per the use of maternal report of fuel type, and cooking and garbage burning practices was sensitive to certain amount of measurement error but was however not likely to be related to the outcome of interest. In summary, it reasonable to assume the exposure assessment reflects reasonably well the exposure conditions during pregnancy.

Although we applied a cross-sectional study design, we were able to collect reasonably valid exposure information from the time period relevant for causation of reduction in fetal growth and therefore our results support a causal relation between the exposures and the outcome of interest. Establishment of temporal relation may be problematic in some research settings with the use of cross-sectional study design. This however should not be a concern in our study because it is clear that exposure to combustion pollutants was present during pregnancy among mothers who cooked with charcoal and those who reported garbage burning at their homes. Also it was increasingly clear from the information collected from the mothers and summarized by the investigators that the choices of cooking fuels and garbage burning activities remained relatively stable in our research settings. It is possible that some women using charcoal during most of the duration of pregnancy might have reported use of LPG as their primary fuel, but this information bias would rather tend to underestimate the true effect. We were not able to undertake air quality measurements in homes of the mothers. The potential for exposure misclassification nonetheless is reduced in our study because the quantitative assessment of exposure to combustion pollutants from charcoal use and garbage burning comprised several types of information collected from participants and summarized by the investigators, including duration of cooking, time spent in cooking area, type of ventilation applied as well as frequency of garbage burning at home and how often mothers were present during combustion.

The study adjusted for the effect of age, social class, marital status and gravidity of mothers, and sex of neonate in the analysis. We had reliable information on gestational age of mothers but did not consider it as a covariate in the analysis based on a recent work by Wilcox and colleagues [15] which reported gestational age as a collider and provided evidence of the likely bias produced by adjustment for gestational age in statistical analysis. We were however unable to examine the effect of other determinants of birth weight such as maternal nutrition and anthropometry, malaria and sexually transmitted infections. IAP exposure during pregnancy is not expected to be dependent on these factors hence the estimated effect of IAP exposure on birth weight is not likely to be confounded by these factors. Maternal smoking is another important determinant of birth weight, but in Ghana only few women smoke. The 2008 Ghana Demographic and Health Survey [16] estimated the proportion of women smoking cigarettes and other tobacco products to be 0.4%. Maternal smoking can therefore not be considered as a serious threat to validity in this study. Cooking fuel was related to social class (p<0.0001), educational level (p<0.0001) and occupation (p<0.0001) of mothers and likely to influence IAP exposure experience during pregnancy. LPG was the primary fuel of high social class mothers with charcoal the preferred fuel of low social class mothers. Uneducated and semi-literate (educated up to junior high school) mothers preferred charcoal with tertiary level educated mothers patronizing LPG. Traders, street vendors, hairdressers and seamstresses had preference for charcoal with office workers preferring LPG. Studies in Ethiopia [17], Cameroun [18] and Kenya [19] also associated household cooking fuel choices with employment, income, educational level and social class of women. We adjusted for the effect of social class but not educational level and occupation of mothers in the analysis due to the well known fact that education and occupation determines social class of an individual, and also the fact they were unrelated to birth weight in our analysis. Controlling for confounding by social class is always problematic especially in our study where more than half (58.8%) of the study participants were low social class. This is due to the strong effects of social class on health outcomes. We do not therefore overrule the possibility of residual confounding by social class in our study, but we think residual confounding does not solely explain our observations on the adverse effects of combustion products from charcoal and garbage burning.

### Synthesis with previous studies

A systematic literature search identified six previous studies conducted in Guatemala [9,10], Zimbabwe [11], Pakistan [12] and India [13,14] that have assessed the relationship between indoor pollution from solid fuel use and birth weight. The study in Guatemala [9] found babies born to mothers who used wood to be 63g lighter than babies born to mothers who used gas or electricity. The other study in Guatemala [10] was a randomized control trial and found infants born to mothers who used open fires (control group) to be on average 89g lighter than infants whose mothers used a chimney stove (intervention group). The study in Zimbabwe [11] found babies born to mothers cooking with wood, dung, or straw to be on average 175g lighter compared with babies born to mothers using LPG, natural gas, or electricity. In the Pakistan study [12], infants born to wood users were on average 82g lighter than infants born to natural gas users. This study also estimated the population attributable risk for LBW explained by wood use to be 24%. The earlier Indian study [13] found infants born to women from households using wood and/or dung as primary cooking fuel to be 104.5g lighter than infants born to mothers from households using biogas or kerosene. This study also reported exposure to biomass fuel to be associated with an adjusted 49% increased risk of LBW. The recent Indian study [14] found children born in households using high pollution fuels (wood, straw, animal dung, crop residues, kerosene, coal and charcoal) to be 73g lighter than those born in households using low pollution fuels (electricity, LPG, natural gas and biogas). A recent meta-analysis [20] of five studies examining this relationship also estimated a reduced mean birth weight of 95.6g (95% CI: 68.5, 124.7) and an increase risk of LBW of 38% (OR = 1.38, 95% CI: 1.25, 1.52) among women exposed to IAP. This study also estimated the population attributable risk for LBW explained by IAP to be 21%.

The findings of our study are consistent with these previous studies albeit our effect estimates were quite larger than any previously reported. The similar results produced from the sensitivity analysis means use of charcoal as cooking fuel and garbage burning at home represents an important threat to optimal fetal growth. We do not by any means imply with the large effect sizes reported that charcoal is a high polluting and more potent fuel than other biomass like wood, dung and crop residue. Garbage burning on the other hand releases dioxins, hazardous chemical substances that have been shown in animal studies to severely impair fetal growth even at low levels of exposure. Studies in human populations have also reported associations of low level dioxin exposure during pregnancy with decreased birth weight [21-24]. Our study and the previous reviewed however did not actually measure the quantity of biomass combusted and the amount of pollutants released for which mothers were exposed. It is therefore reasonable to assume that our study participants might have on average combusted large quantities of charcoal and garbage during pregnancy with the cumulative adverse effect reflected in the large effect estimates reported. The significant exposure-response relationship observed by our study to some extent confirms this assertion. We must however emphasize that unmeasured confounding, and residual confounding by social class as already noted could contribute to the large effect sizes reported in spite of efforts to eliminate this potential confounding from our study.

The studies highlighted however had some limitations which our study was purposely designed to address and strengthen the epidemiological evidence. Firstly, the studies in Zimbabwe [11] and India [14] relied on mothers self-report of child size at birth in respectively estimating birth weight of 47% and 60% of their study infants. This could have resulted in under or over estimation of birth weight of these infants. Secondly, the other studies cited [9,10,12,13] were community-based and obtaining timely measurement of birth weight of infants delivered at home was problematic. The time of measurement in some cases raises doubt about their acceptability as true reflection of birth weight of these infants. A baby's weight can fluctuate within the first week of life with newborns losing up to 10% of their birth weight during the first 3–5 days of life. Some of the studies in an attempt to address this limitation, restricted birth weight analysis to newborns weighed within 48–72 hours after birth. Lastly, majority of households in developing countries use a combination of cooking fuels with those in urban areas especially usually using a combination of polluting and clean fuels as a way of reducing household fuel bills and to at times hasten the preparation of meals. In Ghana for instance, majority of urban households mostly use LPG for preparing sauces, stews, soups and continental meals with charcoal used mainly for preparing staple foods such as banku, fufu and ampesi. Most of the households that use LPG for cooking are also compelled to rely on charcoal when LPG is in short supply. All the studies highlighted collected information on primary cooking fuel of participants without attempting to identify participants using a combination of cooking fuels. This distinction is important for proper quantification of exposure experiences of study participants. For instance, in settings where use of combination of polluting and clean fuels prevails, assessing only primary cooking fuel could result in under or over estimation of exposure. About 19% of the mothers who participated in our study used a combination of charcoal and LPG for cooking.

Our study to the best of our knowledge is the first to examine the contribution of garbage burning at home to the indoor exposure experience of pregnant women, and its relation with birth weight of newborns. Over a quarter (27%) of the mothers studied reported garbage burning in their homes during pregnancy. Garbage burning is a frequent practice in a number of Ghanaian urban households as a way of managing their solid waste. This is because most urban areas especially the secluded and deprived zones are usually not reached with waste collection services. Areas receiving these services are also faced with untimely and irregular service provision. In rural Ghana, the situation is different with younger members of the household tasked with disposing of the household waste at designated sites in the community each morning. The sight of garbage burning in rural households is therefore uncommon.

### Biological plausibility

Burning of charcoal, other solid fuels and garbage emits smoke which contains a number of air pollutants including carbon monoxide (CO) and particulate matter (PM). Inhaled CO and PM impairs fetal growth in two ways; (1) CO combines with hemoglobin to cross the placenta decreasing oxygen supply to tissue which limits the ability of the placenta to transfer nutrients to the fetus, and (2) PM reduces maternal lung function thereby increasing the risk of maternal lung disease, and in turn reducing oxygen delivery to the fetus as well as causing cell damage in the fetus through oxidative stress [25,26]. Impaired fetal growth subsequently leads to reduced or low birth weight [26,27]. Also, reduced oxygen transport across the placenta and fetal uptake due to reduced oxygen supply to the placenta can result in preterm delivery and consequently reduced or low birth weight [27]. The fetus in particular is considered to be highly susceptible to environmental pollutants because of its differential exposure pattern and physiological immaturity [28,29]. The high cell proliferation and changing metabolic mechanisms during the critical phase of fetal development have been identified as the physiological process that renders the developing fetus extremely vulnerable to environmental toxicants [30].

## Conclusions

Our results proved evidence that maternal use of charcoal as a cooking fuel during pregnancy and burning of garbage at home are strong determinants of average fetal growth and risk of LBW. Improving the social status and income levels of women residing in deprived areas, scaling up household waste collection services to cover all areas of urban centers, and increasing LPG production facilities, expanding distribution networks in urban centers, and curbing their competing use in motor vehicles are important in reducing maternal exposures to IAP, and improving birth outcomes in Accra metropolis and other large urban areas in the developing world.

## Abbreviations

CI: Confidence interval; CO: Carbon monoxide; IAP: Indoor air pollution; KBTH: Korle Bu Teaching Hospital; LBW: Low birth weight; LPG: Liquefied petroleum gas; OR: Odds ratio; PM: Particulate matter; RR: Risk ratio; SD: Standard deviation.

## Competing interests

The authors declare that they have no competing interests.

## Authors’ contributions

AKA designed the study with support from GKN and MD, conducted the analysis under the guidance of JJKJ and RQ, and wrote the manuscript with assistance from JJKJ. GKN, MD and RQ reviewed the drafts. All authors read and approved the final manuscript.
